# Cell-Autonomous Alterations in Dendritic Arbor Morphology and Connectivity Induced by Overexpression of MeCP2 in *Xenopus* Central Neurons *In Vivo*

**DOI:** 10.1371/journal.pone.0033153

**Published:** 2012-03-09

**Authors:** Sonya Marshak, Margarita M. Meynard, Ymkje A. De Vries, Adhanet H. Kidane, Susana Cohen-Cory

**Affiliations:** Department of Neurobiology & Behavior, University of California Irvine, Irvine, California, United States of America; University of Nebraska Medical Center, United States of America

## Abstract

Methyl CpG binding protein-2 (MeCP2) is an essential epigenetic regulator in human brain development. Mutations in the MeCP2 gene have been linked to Rett syndrome, a severe X-linked progressive neurodevelopmental disorder, and one of the most common causes of mental retardation in females. MeCP2 duplication and triplication have also been found to affect brain development, indicating that both loss of function and gain in MeCP2 dosage lead to similar neurological phenotypes. Here, we used the *Xenopus laevis* visual system as an *in vivo* model to examine the consequence of increased MeCP2 expression during the morphological maturation of individual central neurons in an otherwise intact brain. Single-cell overexpression of wild-type human MeCP2 was combined with time-lapse confocal microscopy imaging to study dynamic mechanisms by which MeCP2 influences tectal neuron dendritic arborization. Analysis of neurons co-expressing DsRed2 demonstrates that MeCP2 overexpression specifically interfered with dendritic elaboration, decreasing the rates of branch addition and elimination over a 48 hour observation period. Moreover, dynamic analysis of neurons co-expressing wt-hMeCP2 and PSD95-GFP revealed that even though neurons expressing wt-hMeCP2 possessed significantly fewer dendrites and simpler morphologies than control neurons at the same developmental stage, postsynaptic site density in wt-hMeCP2-expressing neurons was similar to controls and increased at a rate higher than controls. Together, our *in vivo* studies support an early, cell-autonomous role for MeCP2 during the morphological differentiation of neurons and indicate that perturbations in MeCP2 gene dosage result in deficits in dendritic arborization that can be compensated, at least in part, by synaptic connectivity changes.

## Introduction

Methyl-CpG binding protein 2 (MeCP2) is a DNA binding protein encoded by a gene located on chromosome Xq28 in humans, and is a protein that is evolutionarily conserved both in structure and in function. Mutations in the *Mecp2* gene have been linked to Rett syndrome in humans (RTT) [1], a severe X-linked progressive neurodevelopmental disorder and one of the most common causes of mental retardation in females. Moreover, *Mecp2* duplication and triplication have been found in males with severe mental retardation [2], [3], [4], [5], indicating that both loss of function and gain in MeCP2 dosage lead to similar neurological phenotypes. In addition to its role in formation and maturation of neuronal circuits, MeCP2 has been implicated in neuronal specification during early embryogenesis in several species, including *Xenopus* [6], [7]. Specifically, studies show that MeCP2 can regulate neuronal precursor cell number by modulating transcription of the neuronal repressor xHairy in the differentiating neuroectoderm of early *Xenopus* embryos [6]. Thus, it is likely that in *Xenopus*, as in other vertebrate species, MeCP2 participates in both early and late aspects of central neuron development.

Rett syndrome is characterized by an early developmental loss of motor skills and language, mental retardation, and autistic behaviors. Significant alterations in dendritic arborization and in dendritic spine number observed in brain samples of Rett patients have led to the hypothesis that developmental defects in synaptic connectivity may be responsible, at least in part, for the deficits caused by this genetic disorder [8]. The onset of RTT in infants occurs before 18 months of age, which corresponds to the time of active synaptogenesis in the brain. Similarly, in rodents, MeCP2 expression in the brain closely correlates with the onset of neuronal maturation and synaptogenesis [9], [10], [11], [12], [13], supporting the notion that MeCP2 may be involved in the formation and/or maturation of synaptic circuits. Studies using mouse models of the disease show that alterations in MeCP2 expression affect dendritic and axon arbor structure, and can also influence both the formation and functional properties of synapses in specific areas of the brain (for review see [14], [15]). Although studies using mouse models of Rett syndrome have proven to be key in identifying neurological phenotypes of the mutations and pinpointing the possible targets of MeCP2, they often involve mutations and/or manipulations affecting a large population of neurons and/or non-neuronal cells [11], [16], [17], [18], [19], [20], [21], [22], [23], [24], [25], [26], [27], [28], thus making it difficult to differentiate between causality and compensatory effects. In this study, we used the *Xenopus* visual system as an *in vivo* model to examine potential cell autonomous effects of altered MeCP2 expression on developing central neurons. Our studies show that MeCP2 is expressed in the visual system of *Xenopus* tadpoles at the time that functional synaptic connections are formed. Moreover, using time-lapse confocal microscopy imaging at the onset of synaptogenesis we demonstrate that overexpression of wild-type human MeCP2 significantly interfered with the elaboration of the dendritic arbor in the individually affected tectal neurons in swimming tadpoles. *In vivo* time-lapse imaging of neurons co-expressing fluorescently-tagged synaptic proteins also revealed that MeCP2 overexpression differentially influences dendritic arborization and postsynaptic site differentiation. Together, our *in vivo* studies support an early, cell-autonomous role for MeCP2 during the morphological differentiation of *Xenopus* central neurons and indicate that increases in MeCP2 gene dosage result in deficits in dendritic growth that can be compensated, at least in part, by an upregulation in synapse number.

## Materials and Methods

### Animals

#### Ethics statement

Animal procedures were approved by the Institutional Animal Care and Use Committee of the University of California, Irvine (Animal Welfare Assurance Number A3416-01). *Xenopus laevis* tadpoles were obtained by *in vitro* fertilization of oocytes from adult females primed with human chorionic gonadotropin (Sigma-Aldrich, St Louis, MO). Tadpoles were raised in rearing solution [60 mM NaCl, 0.67 mM KCl, 0.34 mM Ca(NO3)2, 0.83 mM MgSO4 10 mM HEPES, pH 7.4, and 40 mg/L gentamycin] plus 0.001% phenylthiocarbamide to prevent melanocyte pigmentation. Tadpoles were anesthetized during experimental manipulations with 0.05% tricane methanesulfonate (Finquel; Argent Laboratories, Redmond, WA). Staging was done according to Nieuwkoop and Faber [29].

### RNA extraction, cDNA synthesis and RT-PCR

The midbrains and retinas of stage 40 and 45 *Xenopus* tadpoles were dissected out, individually collected and homogenized in 500 µl ice-cold Trizol (Life technologies, Carlsbad, CA). After chloroform extraction and isopropyl alcohol precipitation, total RNA was dissolved in 25 µl RNase-free dH_2_O, and the total RNA was measured with a DU 530 life science UV/VIS spectrophotometer (Beckman Coulter, Brea, CA). First strand cDNA was synthesized from 0.1 µg total RNA using the Superscript First-Strand Synthesis System for RT-PCR (Invitrogen, Carlsbad, CA) according to the manufacturer's instructions. RT-PCR was performed in a total volume of 25 µl in buffer containing 5 µl cDNA (diluted 5×), 3 mM MgCl2, 0.2 mM dNTPs, 1 U of Platinum high fidelity Taq polymerase (Invitrogen) and 0.3 mM of each primer. Trans-intron primers were designed for MeCP2 (accession No. AF106951) and GAPDH (accession No. U41753) to control for the presence of genomic DNA, with the following sequences: MeCP2, forward 5′-CTTCCTGAAGGCTGGACACG-3′ and reverse 5′-GGAAGTATGCTATAAGCTCAAC-3′; GAPDH, forward 5′-GCTCCTCTCGCAAAGGTCAT-3′ and reverse 5′-GGGCCATCCACTGTCTTCTG-3′. The following thermal profile was used: 95°C for 30 s, 60°C for 30 s and 72°C for 2 min, using a programmable thermal cycler iCycler (Bio-Rad laboratories, Hercules, CA). The PCR products were separated on a 2% agarose gel and visualized with ethidium bromide. No contamination with genomic DNA was observed.

### DNA constructs and plasmid transfections

cDNA coding for human wild type MeCP2 (hMeCP2) was kindly provided by Dr. I. Stancheva (University of Edinburgh, Edinburgh, UK). A hMeCP2-pCS2 plasmid was created by subcloning hMeCP2 cDNA into a pCS2+ expression vector under control of the cytomegalovirus (CMV) promoter. A hMeCP2-IRES-GFP bicistronic plasmid was created by subcloning hMeCP2 cDNA into a pIRES2-EGFP vector (Clontech, Palo Alto, CA). A plasmid coding for a red fluorescent protein variant (pDsRed2, Clontech, Palo Alto, CA) was used to visualize the morphology of tectal neurons transfected with hMeCP2 plasmids. In a first set of studies, the hMeCP2-IRES-GFP construct (MeCP2-IRES group), or a control pIRES2-EGFP construct (IRES-GFP control group) were co-transfected with the pDsRed2 plasmid. Lipofection of a 1∶1 mix of the DsRed2 and IRES plasmids into the brain primordium of stage 20–22 tadpoles was performed in as previously described [30]. Tadpoles were reared until stage 45, when tadpoles with neurons co-expressing both DsRed2 and GFP were selected for imaging. Following imaging, retrospective immunostaining with an antibody against human MeCP2 (Upstate, Lake Placid, NY) was used to further confirm hMeCP2 expression in neurons transfected with hMeCP2-IRES-GFP (see below). In a second set of experiments, expression of a plasmid that encodes the postsynaptic density protein 95 (PSD-95) fused to GFP (PSD95-GFP) [30] was used to visualize postsynaptic specializations in hMeCP2 expressing neurons. Specifically, neuronal precursor cells in brain primordia of stage 20–22 tadpoles were co-lipofected with a mix of DsRed2, PSD95-GFP and hMeCP2-CS2 plasmids (1∶1.5∶2 molar ratio in DOTAP) to follow changes in GFP-labeled postsynaptic sites in hMeCP2 expressing neurons. As controls, neurons were lipofected with only DsRed2 and GFP-PSD95 in stage 20–22 tadpoles. At stage 45, tadpoles with individual DsRed2 and PSD95-GFP double-labeled tectal neurons were selected for time-lapse imaging. Following the last imaging session, retrospective immunostaining was used to confirm expression of hMeCP2 in the PSD95-GFP/DsRed2 positive neurons. Previous electron microscopy and immunohistochemical studies demonstrated that PSD95-GFP specifically localizes to ultrastructurally-identified retinotectal synapses in *Xenopus*, and to synaptic vesicle rich contact sites *in vivo* [30].

### In vivo time-lapse imaging

The branching of individual fluorescently-labeled tectal neurons was followed by confocal microscopy in stage 45 tadpoles. Tadpoles containing individual, clearly distinguishable double-labeled neurons were anesthetized and imaged every 24 h for two days (time points 0 h, 24 h and 48 h). Images were acquired using LSM 5 Pascal confocal microscope with a ×63/0.95 water immersion objective. Optical sections were collected at 1 µm intervals.

### Data analysis

All analysis was performed from raw confocal images without any post-acquisition manipulation or thresholding. Digital three-dimensional reconstructions of DsRed2-labeled dendritic arbors were obtained from individual optical sections through the entire extent of the arbor with the aid of the MetaMorph software (Molecular Devices, Sunnyvale, CA). To characterize the distribution of PSD95-GFP puncta to particular dendritic regions, pixel-by-pixel overlaps of individual optical sections obtained at the two wavelengths were analyzed as described previously [30]. Several morphological parameters were measured for the quantitative analysis of dendritic branching: total branch number, the number of individual branches added and the number of branches remaining from one observation time point to the next (stable branches). Total arbor length was measured from binarized images of the digitally reconstructed dendritic arbors and was converted to microns. To determine dendrite length distribution and the outermost extent of the dendritic arbor, digitally reconstructed dendritic arbors were submitted to Sholl analysis. The length of the longest dendrite was determined as the radius value of the circle with its circumference intersecting with the furthest extending dendrite. The number of 1^st^, 2^nd^, 3^rd^ and 4^th^ order branches in each individual dendritic arbor was measured and used to calculate Dendritic Complexity Index (DCI) as described previously [31]. As outlined above, two sets of experiments were performed, each with its own controls. As controls, neurons co-expressing IRES-GFP/DsRed2 and or PSD95-GFP/DsRed2 were analyzed. No significant difference was found in any of the parameters examined between the two control groups analyzed (IRES-GFP/DsRed2, *n* = 11 vs PSD95-GFP/DsRed2, *n* = 19), or between neurons transfected with the two distinct hMeCP2 expression plasmids (hMeCP2-IRES-GFP, *n* = 11 vs MeCP2-CS2, n = 17). Therefore, data from both control groups and data from both hMeCP2 overexpressing groups was combined for the analysis of branch morphology and dynamics. For the analysis of postsynaptic specializations in expressing neurons, GFP-labeled postsynaptic clusters were counted for each dendritic arbor in the hMeCP2-CS2/PSD95-GFP/DsRed2 transfected neurons and in the corresponding controls. *T-tests* were used for the statistical analysis of data. Results were considered significant as follows: *p≤0.05, **p≤0.005, ***p≤0.0005.

### Immunohistochemistry

For visualization of exogenous hMeCP2 expression, immediately after the last imaging session, tadpoles transfected with DsRed2 and hMeCP2-IRES-GFP plasmids or with DsRed2, PSD95- GFP and hMeCP2-CS2 plasmids were anesthetized and fixed for 2 hr by immersion in 2% paraformaldehyde in 0.1 M phosphate buffer (PB), pH 7.4. Following fixation, brains were removed and postfixed with the same fixative for an additional hour at room temperature. Free-floating brains were preincubated for 1 hr in blocking solution (1.5% normal goat serum and 0.1% Triton X-100 in 0.1 M PB) and incubated overnight with a rabbit polyclonal anti-human MeCP2 antibody (1∶500 dilution; Upstate, Lake Placid, NY). Tissues were then rinsed and incubated with Alexa 633 anti-rabbit antibodies (1∶200 dilution 0.1 M PB; Invitrogen). All images were collected with a LSM 5 Pascal confocal microscope (Zeiss, Jena, Germany) using a ×63/1.4 numerical aperture oil immersion objective, at 1 µm intervals. For visualization of endogenous MeCP2, stage 40 tadpoles were fixed as described above. Twenty-micron cryostat sections were obtained, preincubated for 1 hr in blocking solution (1.5% normal goat serum and 0.1% Triton X-100 in 0.1 M PB) and incubated overnight with rabbit polyclonal anti-MeCP2 antibody (1∶100 dilution; Upstate, Lake Placid, NY) together with a monoclonal antibody against the Vesicle Associated Membrane Protein-2 (VAMP-2) (1∶1000 dilution, QED Bioscience Inc, San Diego, CA). Following washes, sections were incubated with anti-rabbit Alexa 488 and anti-mouse Alexa 568 secondary antibodies (Invitrogen; 1∶500 dilution).

## Results

### MeCP2 is expressed in the developing visual system of *Xenopus* tadpoles

**Figure 1:**
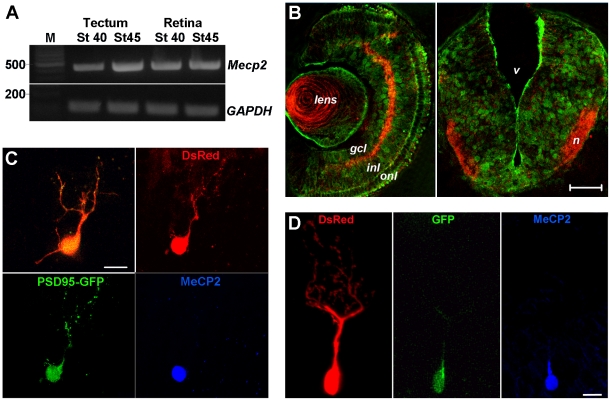
Expression of MeCP2 in the developing *Xenopus laevis* visual system. (**A**) Endogenous expression of *Xenopus* MeCP2 mRNA in the tectum and retina of stage 40 and stage 45 *Xenopus* tadpoles is shown by the RT-PCR reaction products. A single band of the expected molecular weight was observed. Expression of the housekeeping gene *x*-GAPDH is also shown for comparison. DNA molecular weight markers are shown to the left (M, in base pairs). (**B**) MeCP2 protein expression in the retina and optic tectum of Stage 40 tadpoles. *Left panel:* MeCP2 immunopositive cells (green) are localized to the ganglion cell layer (*gcl*) and inner nuclear layer (*inl*) of the developing retina. The retinal synaptic layers are shown by the immunostaining with an antibody to VAMPII (*red*). *Right panel:* Coronal section of a stage 40 tadpole at the level of the optic tectum shows MeCP2 expression in neurons (*green*) close to the tectal neuropil (*n*), which is visualized by VAMPII immunostaining (*red*). V = ventricle. Scale bar = 500 µm. (**C, D**) Transfection with human wild-type hMeCP2 constructs was used to alter expression of MeCP2 in postmitotic *Xenopus* tectal neurons at the onset of synaptic differentiation. **C**) Expression of wt-hMeCP2 was confirmed in triple transfected neurons co-expressing DsRed2, PSD-95-GFP and wt-hMeCP2 as illustrated here by the overlaid live confocal image (overlay), and the red (DsRed2) and green (PSD95-GFP) fluorescence as well as the MeCP2 immunofluorescence (blue) after fixation. **D**) Tectal neuron transfected with DsRed2 and a wt-hMeCP2-IRES-GFP plasmid. Live confocal imaging shows colocalization of DsRed2 (*red*) and GFP (*green*) in the nucleus, cell body, and primary dendrite. Retrospective immunostaining with an antibody directed to human wild-type MeCP2 shows the localization of the MeCP2 protein to the nucleus and proximal portion of the primary dendrite (*blue*). Scale bar for C, D = 10 µm.

Here, we used *Xenopus* as an *in vivo* model system in which manipulations in gene expression can be spatially and temporally controlled to examine the consequence of altered MeCP2 expression during the morphological maturation of central neurons. MeCP2 is expressed in the neuroectoderm of *Xenopus* embryos, where it plays an essential role in early neurogenesis by controlling the expression of proteins downstream of proneural genes [6]. To determine if MeCP2 expression is maintained in *Xenopus* central neurons at the time they differentiate and establish synaptic connections, we analyzed MeCP2 mRNA expression in the visual system of swimming tadpoles by RT-PCR. We found that MeCP2 mRNA is expressed both in the retina and optic tectum of stage 40 and stage 45 tadpoles (Fig. 1A), at the time that retinal ganglion cell axons project to and establish functional synaptic connections with their target neurons in the optic tectum [32], [33], [34]. Immunostaining with an anti-human MeCP2 antibody that, although weak, shows cross-species immunoreactivity with *Xenopus* confirmed MeCP2 protein expression in neurons in the retina and optic tectum at these developmental stages (Fig. 1B). Thus, endogenous MeCP2 is expressed by *Xenopus* central neurons at the time they begin to differentiate and receive functional synaptic inputs.

### Overexpression of hMeCP2 interferes with tectal neuron dendritic arborization

**Figure 2:**
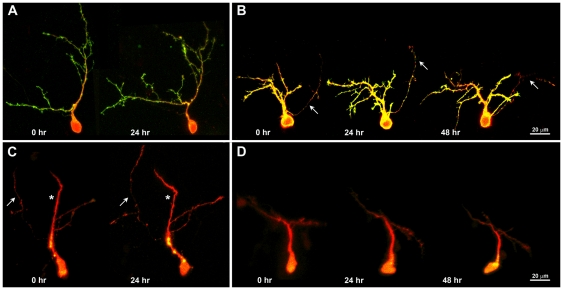
Expression of hMeCP2 influences tectal neuron dendritic branching. (**A, B**) Sample tectal neurons expressing DsRed2 (*red*) together with GFP (*green*; IRES-GFP construct) from stage 45 *Xenopus* tadpoles illustrate the morphologies and dynamics of tectal neuron dendritic branching over time. (**C, D**) Tectal neurons expressing DsRed2 (*red*) and wt-hMeCP2-IRES-GFP (*green*) in stage 45 *Xenopus* tadpoles illustrate the effects of MeCP2 overexpression on dendritic morphology and branch dynamics. In these confocal projections, GFP expression (*yellow*; green and red overlay) confirms the expression of wt-hMeCP2. The asterisks mark a primary dendrite and axons are demarcated by the arrows. Scale bar = 20 µm.

To examine the impact of altered MeCP2 expression during the morphological and synaptic maturation of central neurons, we overexpressed wild-type human MeCP2 cDNA in tectal neurons in live *Xenopus* tadpoles. Targeted co-transfection of plasmids into the developing midbrain of young tadpoles allowed us to increase MeCP2 levels in single tectal neurons as they begin to differentiate, and to examine their morphology at the time they actively branch and establish synaptic contacts in intact tadpoles (stage 45). Dendritic arbor morphology was visualized by expressing DsRed2 together with hMeCP2 (see Methods and Fig. 1 C, D and Fig. 2). An IRES-GFP expression plasmid (hMeCP2-IRES-GFP) was used to express hMeCP2 and to confirm its expression *in vivo* by the independent expression of the GFP marker (Fig. 1 D, Fig. 2 C, D). Alternatively, in a second set of experiments, an expression plasmid coding for wt-hMeCP2 alone was co-transfected with a plasmid coding for GFP-tagged postsynaptic density protein PSD-95 (PSD-95-GFP) and the DsRed2 plasmid. The latter manipulation allowed us to visualize GFP-labeled postsynaptic specializations in a subset of neurons expressing hMeCP2 (Fig. 1C). Retrospective immunostaining with an anti-human MeCP2 antibody confirmed expression of hMeCP2 in the triple (DsRed2/PSD95-GFP/hMeCP2), and double (DsRed2/hMeCP2-IRES-GFP), transfected neurons (Fig. 1 C, D).

**Figure 3:**
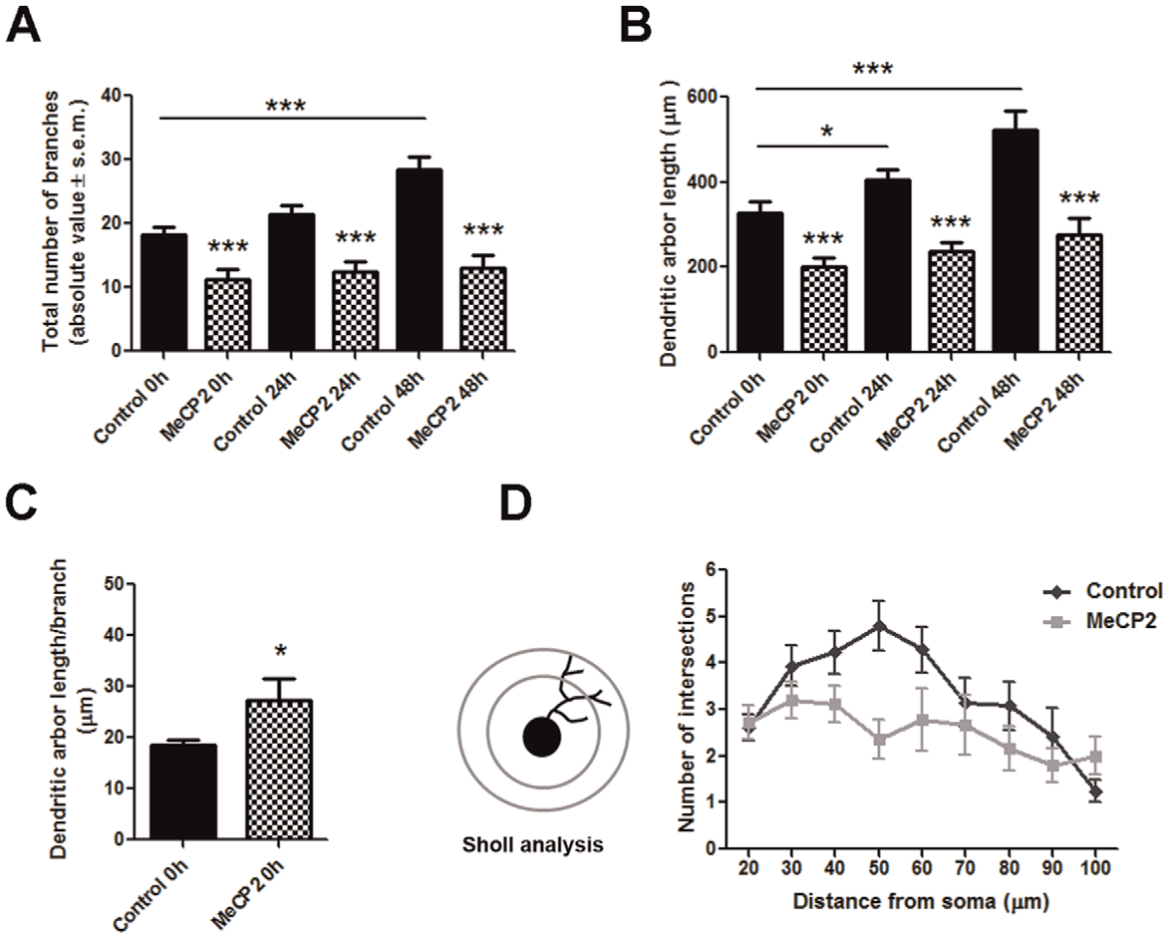
Quantitative analysis of changes in dendritic arbor morphology induced by overexpression of wild-type hMeCP2. (**A**) Total number of branches in tectal neurons of stage 45 *Xenopus* tadpoles at the initial observation time point, and 24 and 48 hours after initial imaging. Note that control neurons increased their total number of branches over a 48 hr period, while wt-hMeCP2 expressing neurons had significantly fewer branches and failed to increase branch number over time. (**B**) Total dendritic arbor length remained significantly lower in MeCP2 overexpressing neurons, while control neurons increase their total dendritic arbor length in every 24 hr observation interval. (**C**) A relative measure of dendritic segment length, calculated as the ratio of total arbor length by total branch number, shows that on average branches in hMeCP2-expressing neurons are longer than in controls. (**D**) *Left*; Sholl analysis was used to determine the number of dendritic crossings in MeCP2 overexpressing and control neurons at 0 hours as measure of dendrite morphology and length. Note that while the maximal extent of the dendritic arbor is similar in hMeCP2-expressing neurons and controls, hMeCP2-expressing neurons have a more uniformly distributed pattern of dendrite lengths. Significance * p≤0.05; ** p≤0.005, ***p≤0.001.

*In vivo* confocal microscopy imaging of tectal neurons expressing hMeCP2 revealed that MeCP2 overexpression significantly altered the morphological differentiation of neurons. At stage 45, tectal neurons expressing hMeCP2 possessed significantly fewer dendrites than control neurons in tadpoles at the same developmental stage (Fig. 2, Fig. 3A). On average, tectal neurons expressing hMeCP2 possessed significantly fewer dendritic branches than controls (Control 18.17±1.23 total branches n = 30 neurons, one neuron per tadpole, MeCP2 11.32±1.45 total branches, n = 28 neurons, one neuron per tadpole; *p* = 0.0007, Fig. 3A). To further determine the impact of MeCP2 overexpression on dendritic branching, neurons were imaged again 24 and 48 hours after the initial time point. Tectal neurons become morphologically more elaborate over time by the dynamic addition and retraction of dendritic branches (see Fig. 2 A, B and also [30], [33]). These dynamic changes in dendritic branching result in arbors with significantly more branches over a 48 hour period (Fig. 3A). Expression of hMeCP2 in single tectal neurons, in contrast, significantly interfered with dendritic arbor growth (Fig. 2 C, D, Fig. 3A). No significant change in total branch number was observed in MeCP2 overexpressing neurons between any of the observation intervals while in controls, a close to 50% increase in branch number was observed in a 48 hour period (Controls; 18.17±1.23 total branches at 0 hr, 28.50±1.9 at 48 hr, *p* = 0.0001; MeCP2 11.32±1.45 branches at 0 hr, 13.07±1.95 at 48 hr, *p* = 0.47; Fig. 3A). Thus, hMeCP2-expressing neurons remained relatively simple over time.

Similar to its effect on branch number, total arbor length was significantly lower in the hMeCP2- expressing neurons than in control neurons in stage 45 tadpoles (Control 328.7±26.68 µm, MeCP2 202.0±18.99 µm, *p* = 0.0004; Fig. 3B). Total dendritic arbor length remained significantly lower in the hMeCP2-expressing neurons 24 and 48 hours after the first imaging when compared to control neurons (Fig. 3B). The length of the dendritic arbor of hMeCP2-expressing neurons increased over time, a difference that almost reached significance within 48 hours after initial imaging (dendrite arbor length 202.9±18.99 µm at 0 hr; 278.2±38.0 µm at 48 hr, p = 0.0551; Fig. 3B). The observation that MeCP2 overexpression negatively affects branch number more than arbor length suggested that overall, the extended dendritic branches in hMeCP2-expressing neurons may be longer in length. Indeed, a relative measure of dendritic segment length, calculated as the ratio of total arbor length by total branch number, showed that on average branches were longer in hMeCP2-expressing neurons than in control neurons at the same developmental stage (Control 18.52±1.10 µm/branch at stage 45, t = 0, MeCP2 27.22±4.356 µm/branch at stage 45, t = 0; *p* = 0.0411; Fig. 3C). Sholl analysis also revealed that while dendritic processes were fewer, they were widely distributed along the arbor in the hMeCP2-expressing neurons (Fig. 3D), with the maximal extent of the dendrites in the neurons overexpressing MeCP2 not differing from controls (Control 75.00±5.378 µm at stage 45, t = 0, MeCP2 74.62±7.043 µm at stage 45, t = 0; *p* = 0.968). Thus, the increase in dendrite arbor length but not branch number indicates that although MeCP2 overexpression mainly interfered with dendritic branch initiation, it did not completely interfere with the ability of individual dendrites to lengthen.

**Figure 4:**
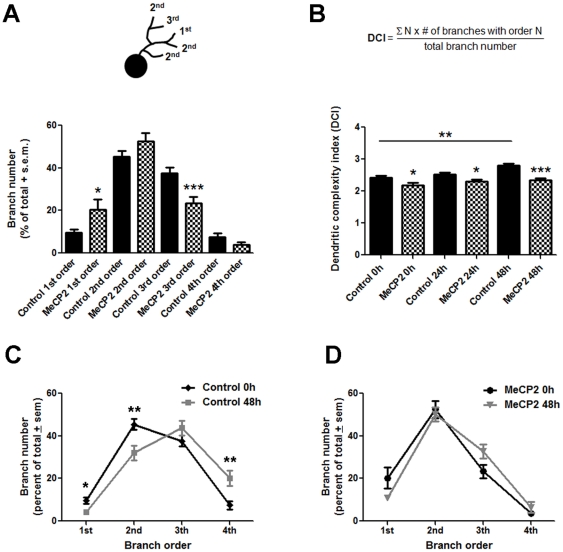
hMeCP2-expressing neurons develop morphologically simple dendritic arbors. (**A**) The complexity of the dendritic arbors in control neurons expressing DsRed2 and in neurons co-expressing DsRed2 and hMeCP2 is exemplified by the proportion of first, second, third and fourth order branches, expressed as percent of their total branch number. Note that MeCP2 overexpressing neurons have proportionately more first order branches but fewer third order branches. (**B**) *Top*; The Dendritic Complexity Index (DCI) provides an additional measure of dendritic morphology. *Bottom graph*; The DCI value for MeCP2 expressing neurons was significantly lower than the value for control neurons at the initial observation time point. Moreover, while control neurons significantly increased their DCI value by 48 h, DCI value for hMeCP2-expressing neurons did not change over time. **C, D**) Branch order distribution at 0 and 48 hours for (***C***) control, and (***D***) hMeCP2-expressing neurons. Note the significant shift in distribution of branches in control neurons, indicating an increase in complexity over time, while no change was observed over a 48 hour period in neurons overexpressing MeCP2. Significance * p≤0.05; ** p≤0.005, ***p≤0.001.

To obtain an additional measure of the effect of MeCP2 overexpression on dendritic branching, we determined the proportion of primary, secondary and tertiary branches in neurons expressing hMeCP2 and compared it to controls. Analysis of branch order distribution at the initial observation time point (stage 45, t = 0) showed that in proportion, hMeCP2-expressing neurons had significantly fewer tertiary branches than controls (3^rd^ order branches: Controls 40.53±3.34%, MeCP2 23.42±3.14%, *p* = 0.0007; Fig. 4A), extending mostly primary and secondary branches (1st order branches: Controls 7.889±1.70%, MeCP2 20.31±4.84%, *p* = 0.045; Fig. 4A). Thus MeCP2 overexpression resulted in neurons with simpler dendritic arbors than controls. To further determine the effect of MeCP2 overexpression on dendritic branching over time, we evaluated dendritic arbor complexity by assigning a topological order to every branch in each individual dendritic arbor and by calculating a Dendritic Complexity Index (DCI) using the formula described in Fig. 4B. DCI provides an additional relative measure of branch order number [31]. At the initial observation time point, MeCP2-expressing neurons possessed a DCI value lower than controls (controls 1.52±0.076, MeCP2 1.26±0.066, p = 0.013; Fig. 4B), indicating a simpler dendritic arbor complexity. Moreover, within 48 hours, control neurons significantly increased their DCI value (DCI controls: t = 0 hr 1.52±0.076, t = 48 hr 1.81±0.069, p = 0.017; Fig. 4B) and branch order number (Fig. 4C), whereas in MeCP2 overexpressing neurons DCI values and branch order number (Fig. 4D) did not change over a 48 hour period. Thus, as a consequence, MeCP2 neurons were simpler in morphology, with fewer high-order branches, but with primary and secondary branches that were normal in length. Together, our data demonstrates that MeCP2 overexpression interferes with the ability of tectal neurons to arborize and to increase the morphological complexity of their dendritic arbor.

### Overexpression of MeCP2 affects new branch addition

**Figure 5:**
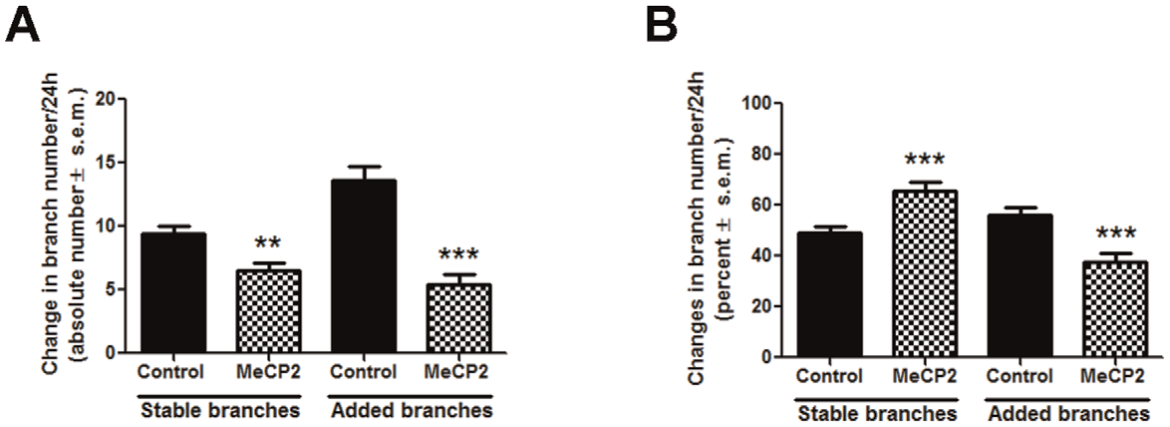
Overexpression of MeCP2 decreases new branch formation in developing tectal neurons but does not interfere with the stability of existing branches. The absolute (**A**) and relative (**B**) number of stabilized and newly added branches in MeCP2 overexpressing neurons compared to controls are shown by the bar graphs. hMeCP2-expressing neurons added significantly fewer new branches than controls during every 24 imaging period (0–24 h and 24–48 h, combined). As percentage, the number of dendritic branches stabilized over a 24 h period is significantly higher in hMeCP2-expressing neurons than controls (***B***), although hMeCP2-expressing neurons had fewer dendritic branches overall (***A***, absolute values; see also ***Fig. 3***). Significance * p≤0.05; ** p≤0.005, ***p≤0.001.

Developing tectal neurons significantly increase dendritic branch number and complexity by stabilizing a large portion of their branches and by adding new branches while also eliminating some of the existing branches [30], [33], [35], [36]. To further examine the effects of MeCP2 overexpression on dendritic branch dynamics we analyzed whether hMeCP2 expression altered the addition, elimination and/or stability of individual dendritic branches over time. Analysis of branch dynamics revealed that hMeCP2-expressing neurons continued to add new branches during every 24 hour imaging interval, although fewer in number than control neurons (Added branches: Control 13.79±1.149 branches/24 h, MeCP2 5.459±0.7833 branches/24 h; *p*<0.0001, Fig. 5A). Similarly, the proportion of added branches (percent of total branches) in hMeCP2-expressing neurons was also significantly lower than in controls (Added branches: Control 57.13±2.75%, MeCP2 37.62±3.51%, *p* = 0.001; Fig. 5B). Time-lapse imaging also revealed that as many branches were eliminated in hMeCP2-expressing neurons as were added within a 24 hour period (MeCP2: 5.459±0.7833 branches added, 4.50±0.762 branches eliminated, *p* = 0.386), while in controls significantly more branches were added than eliminated thus promoting growth (Control: 13.79±1.149 branches added, 10.14±0.846 branches eliminated, *p* = 0.012; for branches eliminated in hMeCP2-expressing neurons vs. controls, *p*<0.0001; not shown graphically). Moreover, analysis of branch dynamics revealed that even though MeCP2 overexpressing neurons possessed relatively fewer branches that were stabilized than control neurons at the same developmental stage (Stable branches: Control 9.429±0.62, MeCP2 6.514±0.58, Fig. 2 and Fig. 5A), in proportion, those existing branches were significantly stable (Control 49.16±2.45%, MeCP2 65.78±3.17%, p<0.0001; Fig. 5B). Consequently, the lower branch addition and elimination rates observed in hMeCP2-expressing neurons indicates that MeCP2 overexpression results in dendritic arbors that are morphologically simpler and less dynamic, but that are able to retain stably those few branches that are initially extended.

### MeCP2 overexpression increases postsynaptic specialization density in tectal neuron dendritic arbors

**Figure 6:**
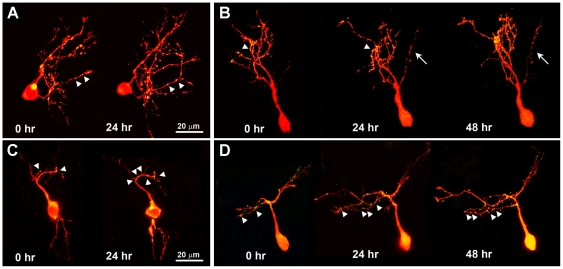
Postsynaptic site differentiation in hMeCP2-expressing tectal neurons. Time lapse confocal images of representative control (***A, B***) and hMeCP2-expressing (***C, D***) tectal neurons co-expressing DsRed2 (*red*) and PSD95-GFP (*green*) in stage 45 *Xenopus* tadpoles illustrate the morphologies and distribution of PSD95-GFP postsynaptic specializations (*yellow puncta*; red and green overlap) on the dendritic arbors. (**C, D**) Tectal neurons co-expressing wild-type hMeCP2 together with DsRed2 and PSD95-GFP show an increase in the density of postsynaptic clusters (*yellow puncta*, arrowheads) over a 24 and 48 h observation period. Axons of tectal neurons are marked by white arrows. Expression of hMeCP2 was confirmed by retrospective immunostaining as in Fig. 1D. Scale bar = 20 µm.

**Figure 7:**
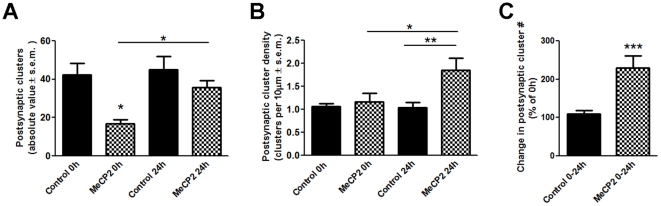
Expression of hMeCP2 differentially influences dendritic branching and postsynaptic site differentiation. The effects of MeCP2 overexpression on postsynaptic specializations in the tectal neuron dendritic arbors are shown by the bar graphs. (**A**) The absolute number of PSD95-GFP postsynaptic clusters in the hMeCP2-expressing neurons is lower than in controls both at 0 and 24 h. (**B**) When normalized per unit arbor length, the density of PSD95-GFP postsynaptic clusters is similar to controls at the initial observation time point but increases significantly more than controls by 24 hours. (**C**) hMeCP2-expressing neurons increase their postsynaptic clusters number by approximately two-fold in 24 hours. Significance * p≤0.05; ** p≤0.005, ***p≤0.001.

Previous studies demonstrate that developing neurons establish synapses in a dynamic way, and that synapse formation coincides with the active elaboration and branching of the neuron's dendritic arbor. To further establish a potential relationship between synapse formation and dendritic elaboration in response to altered MeCP2 expression, we used single cell co-transfection of wt-hMeCP2, DsRed2 and PSD95-GFP expression plasmids in live *Xenopus* embryos. PSD95-GFP specifically localizes to synaptic contact sites *in vivo* and to ultrastructurally identified synapses in tectal neuron dendrites [30]. Thus, this allowed us to visualize postsynaptic specializations in single neurons overexpressing MeCP2 in an otherwise intact brain (Fig. 6). Because dendritic arbors in MeCP2 expressing neurons were overall simpler, the total number of postsynaptic specializations in MeCP2 neurons was significantly lower than controls at stage 45, the initial observation time (t = 0 hr: Control 53.43±10.12; MeCP2 16.83±3.04, *p* = 0.008, Fig. 6, Fig. 7A). Our imaging studies revealed, however, that although hMeCP2-expressing neurons possessed fewer dendritic branches, the density of postsynaptic specializations, measured as the number of PSD95-GFP puncta per µm of arbor length, was similar to controls (Control 1.059±0.06, MeCP2 1.169±0.186, *p* = 0.531, Fig. 7B). To support the observation that MeCP2 overexpression indeed influences postsynaptic density, we compared the morphology and PSD95-GFP cluster density in hMeCP2-expressing neurons (stage 45) with that of control neurons of stage 43 tadpoles (24 hours earlier) that more closely matched the maturational state of neurons overexpressing MeCP2 at stage 45. Postsynaptic specialization density in hMeCP2-expressing neurons at stage 45 was significantly higher than that of control tectal neurons in younger tadpoles (Stage 43 controls 0.7939±0.04 PSD95-GFP puncta per µm, Stage 45 MeCP2; 1.169±0.1869 PSD95-GFP puncta per µm, *p* = 0.0145). Tectal neurons of stage 43 tadpoles possessed dendritic arbors with branch numbers and total dendrite arbor length similar to the hMeCP2-expressing neurons at stage 45 (Stage 43 control; branch number 11.83±0.8717, total arbor length 233.4±14.53, *n* = 18 neurons, see Fig. 3A), therefore postsynaptic specialization density was significantly higher in MeCP2 expressing neurons than their maturation matched controls. Moreover, time-lapse imaging revealed that the number and density of postsynaptic specializations in MeCP2 overexpressing neurons increased significantly over time (PSD95-GFP cluster number: *t* = *0 hr* 16.83±3.04; *t = 24 hr* 35.83±5.22; *p* = 0.0104; *t = 48 hr* 41.00±9.29, *p* = 0.0149, Fig. 7 A, B), and at a rate higher than their age-matched controls (stage 45; Fig. 7C). This significant increase in PSD95-GFP puncta in the MeCP2 expressing tectal neurons as they develop over time suggests the existence of mechanisms that take effect to compensate for a morphologically simpler dendritic arbor.

## Discussion

In this study, we used the developing *Xenopus* retinotectal system as an *in vivo* model to examine cell autonomous effects of MeCP2 expression during the morphological maturation of individual central neurons in an otherwise intact brain. Our results show that a gain in MeCP2 function, achieved by the expression of wild-type human MeCP2 above the endogenous *Xenopus* MeCP2 expression, interfered with the morphological differentiation of tectal neuron dendritic arbors while simultaneously increased the formation of postsynaptic specializations on the few dendrites that developed and remained stable. These changes in dendritic arbor elaboration and postsynaptic differentiation occurred at the time when neurons begin to branch and differentiate, and were sustained throughout the imaging period. Therefore, cell-specific alterations in MeCP2 expression are sufficient to impact the connectivity and function of neurons in the developing, living brain.

Mutations in the MeCP2 gene, loss of gene function, as well as increased MeCP2 gene copy number have been associated with neurodevelopmental defects that lead to Rett syndrome and mental retardation [1], [2], [3], [4], [5]. Anatomical post-mortem data in humans reveal that alterations in MeCP2 expression lead to changes in synaptic number and in neuronal morphology in affected brains [8], [37]. Both transgenic and knockout mouse models have provided important information for understanding how MeCP2 miss-expression affects brain development and function [16], [17], [18], [19]. However, confounding or opposing results have been obtained amongst some studies that seem to depend on the particular mutation or analysis performed [11], [20], [21], [22], [23], [24], [25], [26], [27], [28]. For example, studies have reported a decrease in dendritic branch complexity upon MeCP2 downregulation *in vivo* and in culture [11], [23], [28], [38] while others show no effect of decreased MeCP2 expression on neuronal complexity [21], [25]. Interestingly, both downregulation and overexpression of MeCP2 in knockout and transgenic mice have been shown to impact dendritic morphology of hippocampal neurons in similar ways, suggesting that threshold levels of MeCP2 expression are required for the maintenance of dendritic arbor structure [28]. Here, by examining the early, dynamic effects of MeCP2 overexpression in single *Xenopus* central neurons *in vivo* we demonstrate that MeCP2 regulates different aspects of neuronal maturation and of synaptogenesis in a cell autonomous way. The observation that individual *Xenopus* tectal neurons overexpressing MeCP2 are morphologically less mature at the time of active dendritic elaboration than control neurons at the same developmental stage is consistent with observations that MeCP2 overexpression negatively influences the branching of mouse hippocampal neurons in culture [28]. Tectal neurons expressing wt-hMeCP2 had significantly less branches than controls and remained simple over the course of 48 hours of imaging. However, even though neurons overexpressing MeCP2 failed to increase dendritic branch complexity over time, the extended dendrites retained the ability to elongate. Thus, in the developing *Xenopus* visual system, altered level of MeCP2 expression and function impact the morphological maturation of tectal neurons by interfering with dendrite branching but not dendrite growth.

Effects of altered MeCP2 expression on synapse density and function have been described for multiple neuronal populations in animal model systems [15], [39], [40]. Observations of altered synaptic connectivity in models of Rett syndrome correlate with observations of decreased dendritic spine number and loss of spines in the cortex in brains of subjects with RTT [8], [37], [41], [42]. In our study, we also examined the effects of MeCP2 overexpression on the ability of central neurons to form synaptic specializations on their dendrites. Our results demonstrate that even though dendritic arbors in individual neurons expressing hMeCP2 were less branched, the density of postsynaptic specializations, as visualized by the punctate accumulation of PSD-95-GFP, remained similar to those observed for control neurons in the intact brain. Importantly, time-lapse imaging revealed that neurons overexpressing MeCP2 increased the rate at which postsynaptic specializations continued to form. These results therefore indicate that gain of MeCP2 function interferes with dendrite initiation and branching, thus resulting in deficits in overall dendritic growth, but that these deficits in connectivity can be compensated, at least in part, by an increase in synapse number on the stunted dendrites. In agreement with our observation that synapse density is gradually increased in tectal neurons overexpressing MeCP2 are studies demonstrating that in hippocampal neurons of transgenic mice in which MeCP2 dosage is doubled, the density of glutamatergic synapses is increased even though dendritic branching and length are unchanged [21]. Thus, consistent with observations in mammals [21], MeCP2 influences the synaptic maturation of neurons independent of its effects on dendritic arbor morphology and growth, allowing for compensatory mechanisms that adjust synaptic inputs to dendrite arbor geometry to maintain structural homeostasis [43].

In the *Xenopus* visual system, MeCP2 is expressed in both the retina and the optic tectum at the time of active retinotectal circuit formation and at the time when functional synaptic connections are formed. The observation that MeCP2 is expressed in the retinotectal system of this species is consistent with previous findings demonstrating high MeCP2 expression in early *Xenopus* embryos [6] and in the embryonic and adult zebrafish brain [7]. Hence, MeCP2 can play multiple roles during the formation and/or maturation of neuronal circuits in the vertebrate nervous system. In *Xenopus*, MeCP2 can regulate neuronal precursor cell number by modulating transcription of the neuronal repressor xHairy in the differentiating neuroectoderm early during embryogenesis [6]. MeCP2 is also considered to be a regulator of global chromatin architecture in the cell, where it acts as both positive and negative transcriptional regulator of hundreds of target genes [44], [45], [46]. The genes putatively activated by MeCP2 code for proteins involved in neuronal development, synaptic transmission and neurotransmitter transport. Moreover, neuronal activity can further modulate MeCP2 binding affinity to the DNA [47]. One of the well established downstream targets of MeCP2 is the BDNF gene [28], [48], [49]. BDNF expression can be upregulated by overexpression of MeCP2 and down-regulated in MeCP2-null animals [44], [50]. BDNF knockout mice share many overlapping phenotypes with MeCP2 knockout mice, including smaller brain size, reduced dendritic arbor complexity and impaired synaptic plasticity. Moreover, BDNF overexpression in the MeCP2 mutant brain reverses some deficits observed in MeCP2 mutants [51], [52], suggesting that MeCP2 affects neuronal functions in part through a BDNF dependent mechanism. Our previous studies show that BDNF plays crucial roles during *Xenopus* retinotectal circuit development, promoting synaptogenesis and retinal ganglion cell axon arbor branching and maintenance [31], [53], [54]. Here, we demonstrate that MeCP2 participates in morphological and synaptic development of tectal neurons in a cell autonomous way. While manipulations in BDNF levels or BDNF receptor signaling do not directly influence dendritic arborization of tectal neurons [30], [31], it is possible that MeCP2 influences tectal neurons and retinotectal circuit development, at least in part, through a BDNF-mediated mechanism as tectal neurons express BDNF [55]. Consistent with this possibility, morpholino antisense oligonucleotide downregulation of MeCP2 expression resulted in altered BDNF expression in the *Xenopus* eye and midbrain, but did not influence the expression of the BDNF receptor TrkB (A.H.K. and S.C-C, unpublished).

Our finding that MeCP2 impacts neuronal cytoarchitecture and connectivity in the amphibian brain indicates that MeCP2 function during neuronal development is conserved. How MeCP2 dysfunction impacts the morphological development of neurons, however, seems to depend on the type of neuron that is affected, the developmental stage at which MeCP2 expression and function are altered, and the particular brain region and species analyzed [15]. A large body of evidence also indicates that MeCP2 gene copy number, the type and penetrance of the identified mutations (partial or complete loss-of function), and whether MeCP2 acts cell- or non-cell-autonomously, are important determinants for MeCP2 function [56]. Observations that overexpression of wild type MeCP2 in cultured cortical neurons increases dendrite branching and dendritic processes length while expression of a mutant form of MeCP2 results in highly branched dendrites that are short in length [24] indicate that the gain and loss of MeCP2 function may produce similar phenotypes, and support the notion that the precise levels of MeCP2 are key determinants during the morphological development of neurons. Our observation that expression of a mutant form of human MeCP2 (MeCP2 R168X mutation lacking the transcription repression domain of the protein) above endogenous *Xenopus* MeCP2 levels did not influence tectal neuron dendritic morphology or growth (S. Marshak, data not shown) while overexpression of wild-type hMeCP2 significantly impacted dendritic morphology, is also consistent with the idea that the levels of functional MeCP2 protein within a neuron are important for proper development and function, and that non-functional mutations cannot substitute for endogenous MeCP2 during dendritogenesis. Increased MeCP2 expression can therefore have significant functional consequences for the morphological and functional development of *Xenopus* central neurons as in other vertebrate species. Indeed, increased MeCP2 copy number caused by duplication and triplication of part of the Xq28 chromosome carrying the MeCP2 gene causes a neurodevelopmental disorder in boys that is characterized by severe mental retardation. [4], [5].

That MeCP2 can exert both cell-autonomous and non-cell-autonomous effects on neurons has recently been suggested by studies that analyzed mouse models of RTT with targeted mutations of MeCP2 [57], [58], [59]. Consistent with a cell-autonomous role for MeCP2 is also evidence that *Mecp2*-null neurons develop dendritic arborization defects when transplanted to a wild-type cortex [60]. Evidence demonstrating that mosaic expression of MeCP2 in brains of heterozygote female mutant mice results in wild-type neurons with dendrites that are less branched also supports non-cell autonomous effects of the MeCP2 mutation [61]. Moreover, evidence that astrocytes and microglia also express MeCP2 and that in the absence of MeCP2 expression glia can influence the morphological development of neurons point to a glial-mediated mechanism by which MeCP2 influences neuronal development [62]. Collectively, our *in vivo* imaging studies reveal a novel cell-autonomous effect of MeCP2, where increased expression of MeCP2 significantly impacts the morphological differentiation of affected central neurons and in turn influences their synaptic connectivity. It is also possible, however, that MeCP2 expression in the *Xenopus* central nervous system may affect neuronal development and function in both cell-autonomous and non-cell-autonomous ways as is the case in the mammalian brain [61]. Potential non-cell autonomous roles for MeCP2, and the mechanisms and interactions between MeCP2 function and BDNF during retinotectal circuit formation are intriguing possibilities that remain open for investigation.

In conclusion, by using an *in vivo* model system in which manipulations can be spatially and temporally controlled to examine the consequence of altered MeCP2 gene expression our studies demonstrate that MeCP2 exerts direct, cell-autonomous effects during the morphological and synaptic differentiation of *Xenopus* central neurons. These studies support the idea that MeCP2 gene dosage is important for proper neuronal differentiation and maintenance, as alterations in MeCP2 expression can lead to significant early defects in the growth and differentiation of individual neurons, thus impacting their connectivity in an otherwise intact brain.
